# Global Potential to Increase Soil Carbon Storage by Reducing Rotational Fallow in Semiarid Regions

**DOI:** 10.1111/gcb.70672

**Published:** 2025-12-26

**Authors:** Chang Liang, Bert VandenBygaart, Stephen Ogle, Douglas MacDonald, Gabriel Dias Ferreira

**Affiliations:** ^1^ Pollutant Inventories and Reporting Division Environment and Climate Change Canada Gatineau Quebec Canada; ^2^ Ottawa Research and Development Centre, Agriculture and Agri‐Food Canada, Central Experimental Farm Ottawa Ontario Canada; ^3^ Natural Resource Ecology Laboratory Colorado State University Fort Collins Colorado USA

**Keywords:** bare‐fallow, carbon storage, soil organic carbon, soil type, summer‐fallow, texture

## Abstract

Intensification of cropping systems improves crop productivity and soil organic carbon (SOC) storage by maintaining or enhancing existing SOC stocks. We compiled published data on SOC changes in agricultural soils globally from experiments evaluating the impact of bare‐fallow reduction to determine the change in SOC storage that results from management change. Overall, the intensification of cropping systems by eliminating fallow led to an average increase in SOC stocks that were 3.2 (±0.3) Mg C ha^−1^ than in cropping systems with bare‐fallow. To account for variation in fallow frequency among study sites, we estimated the SOC change on a per year of fallow reduction basis and found the difference in SOC stocks was 443 (±34) kg C ha^−1^ for each year of fallow reduction. Soil texture influenced the amount of SOC change, with average differences of 552 (±85), 406 (±38), and 430 (±92) kg C ha^−1^ yr^−1^ for fallow reduction in fine‐, medium‐, and coarse‐textured soils, respectively. The rate of SOC storage declined over time with SOC increases of 615 (±74), 433 (±45), and 360 (±60) kg C ha^−1^ associated with fallow reduction for < 10 years, 11 to 20 years, and > 21 years, respectively. Soil type and aridity index had an impact on SOC storage when comparing crop systems with and without fallow. Countries with significant amounts of bare fallow could promote intensification of cropping systems by reducing bare fallow as part of their nationally determined contributions to the Paris Agreement. Canada has reduced the annual area of fallow from 1990 to 2022, resulting in a cumulative gain of SOC storage of 66.3 Mt C. Based on global statistics of annual fallow area, a significant reduction in this practice is feasible on a global scale with cumulative changes in SOC storage of 0.54 Gt C for a period of 20 years.

## Graphical abstract

Intensification of cropping systems improves crop productivity and soil organic carbon (SOC) storage by maintaining or enhancing existing SOC stocks. We compiled published data on SOC changes in agricultural soils globally from experiments evaluating the impact of bare‐fallow reduction to determine the change in SOC storage that results from management change. To account for variation in fallow frequency among study sites, we estimated the SOC change on a per year of fallow reduction basis and found the difference in SOC stocks was 443 (±34) kg C ha^−1^ for each year of fallow reduction.

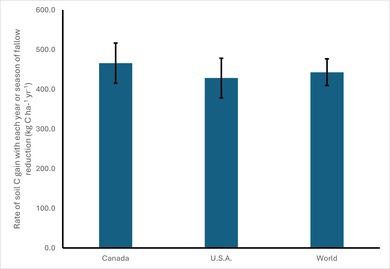

## 1 Introduction

The quantity of organic carbon contained in the world's soils is estimated at about 1500 Gt to a depth of 1 m, about two times that in atmospheric CO_2_, and is of importance in the global C cycle (Powlson et al. 2018). Minasny et al. (2017) conducted a survey to determine the potential for enhancing soil organic carbon (SOC) storage from 20 regions in the world and reported that annual C sequestration rates globally under best management practices could increase SOC by 4‰ or greater. To the contrary, Powlson et al. (2018) argued that the 4‰ annual rate of SOC increase (Minasny et al. 2017) is unachievable in most agricultural situations and cannot be regarded as a major contributor for efforts to limit climate change through global efforts, such as the Paris Agreement. However, others have argued that there is potential for meaningful levels of SOC storage that can be achieved in agricultural soils as a natural climate solution (NCS) while producing food and fibre for an ever‐growing population (Paustian et al. 2016; Lu et al. 2018; Roe et al. 2021; Lal 2022).

In recent years, considerable efforts have been placed on NCS, a suite of approaches to improve management and/or restore forests, grasslands, agricultural soils, and wetlands that can provide climate mitigation beyond business as usual. Some studies estimate that full implementation of all cost‐effective natural climate solutions can provide up to one‐third of the global mitigation needed in 2030 to keep warming below 2°C (Griscom et al. 2017). For example, NCS could mitigate up to 21% of net annual emissions in the United States (Fargione et al. 2018) and 78.2 Mt. CO_2_e yr^−1^ in Canada at a price of CAD 50 dollars per tonne CO_2_ or less (Drever et al. 2021). However, many of the agricultural NCS practices proposed may potentially have yield penalties and/or increased cost or labour implications for farmers and therefore may only be applicable to a narrow range of production systems that will result in real carbon sequestration or emission reduction.

In semi‐arid regions, such as the Central and Northern Great Plains of North America, summer fallow is a traditional farming practice in which farmers do not plant their fields during the summer season to reduce nutrient and water losses from the soil (Campbell et al. 2005). This practice was and is still used in rainfed farming systems with extensive use of mechanical tillage for weed control during the fallow year (Curtin et al. 2000). Cropping sequences such as spring wheat–fallow (WF) can reduce residue inputs to the soil, while relatively high soil disturbance through conventional tillage can exacerbate losses of SOC by increasing microbial decomposition that returns C to the atmosphere. However, there are other ways to conserve moisture in the soil, such as conservation tillage that limits evaporation of water from the soil surface, thereby reducing the need for a fallow year in the crop systems and promoting soil C sequestration (Baumhardt et al. 2015; Hansen et al. 2012). Further, reductions of fallow may increase the overall yield and, as such, have positive impacts on global food production.

No‐till and minimal tillage systems have allowed producers in the semiarid Great Plains of North America to intensify the frequency of cropping when compared with the traditional crop‐fallow system. Cihacek and Ulmer (1995) point out that more intensive cropping systems than crop‐fallow along with reduced tillage are needed to prevent the loss of SOC from soils in the U.S. Great Plains. The fallow period represents a time of high microbial activity and decomposition of soil organic matter without input of crop residue (Campbell et al. 2005). Fallow also represents a time when the soil is susceptible to wind erosion, which is another major loss mechanism for SOC (Haas et al. 1974).

It is established that eliminating or reducing the frequency of fallow increases C input within cropping systems, but large variation in either net change in storage or average rate of gains in SOC has been found in the literature, mainly reflecting differences in fallow frequency, soil types, and duration of field studies (Campbell et al. 2005; McConkey et al. 2003). For instance, Grant et al. (2001) compared a single fallow followed by 4 years of wheat (FWWWW) to a fallow‐wheat (FW) system for a period of 63 years in Alberta, Canada, and reported a net increase in SOC storage of 10 Mg C ha^−1^ or 160 kg C ha^−1^ yr^−1^ in a medium‐textured soil. Campbell et al. (2005) compared continuous wheat (ContW) with FW in the Red River region of Manitoba for a period of 37 years and reported a net change of 34.8 Mg C ha^−1^ or 940 kg C ha^−1^ yr^−1^ in a fine‐textured soil of Udic Boroll (Canadian soil taxonomy: Black Chernozem). Higashi et al. (2014) also reported a significant gain of 9.5 Mg C ha^−1^ for a period of 9 years by comparing continuous soybean with soybean‐fallow in an annual double cropping system in Japan or 1060 kg C ha^−1^ season^−1^ in a medium‐textured Volcanic Andosol. In contrast, many studies have shown that SOC is only 2.1 to 3.7 Mg ha^−1^ higher in continuous wheat than in fallow wheat (Campbell, Biederbeck, et al. 1991; Campbell, Bowren, et al. 1991; Campbell and Zentner 1993; Bremer et al. 1994), and there have even been losses of SOC observed with more intensive cropping systems (Sherrod et al. 2003; Halvorson et al. 2016; Curtin et al. 2000).

The objective of our study is to review the experimental evidence and quantify the effect of cropping system intensification on SOC storage. We are analyzing changes in SOC storage and not explicitly determining if the effect is a reduction in the loss of C from soils or an explicit increase in SOC that is removing CO_2_ from the atmosphere. Regardless of the effect, our study is assessing a change in the SOC pool that will reduce the amount of CO_2_ emissions from soils or increase the amount of CO_2_ uptake, thereby reducing CO_2_ concentrations in the atmosphere. In addition, we evaluate key factors that control the change in SOC as related to soil texture, climatic variables, soil type, and duration of field studies.

## 2 Materials and Methods

**FIGURE 1:**
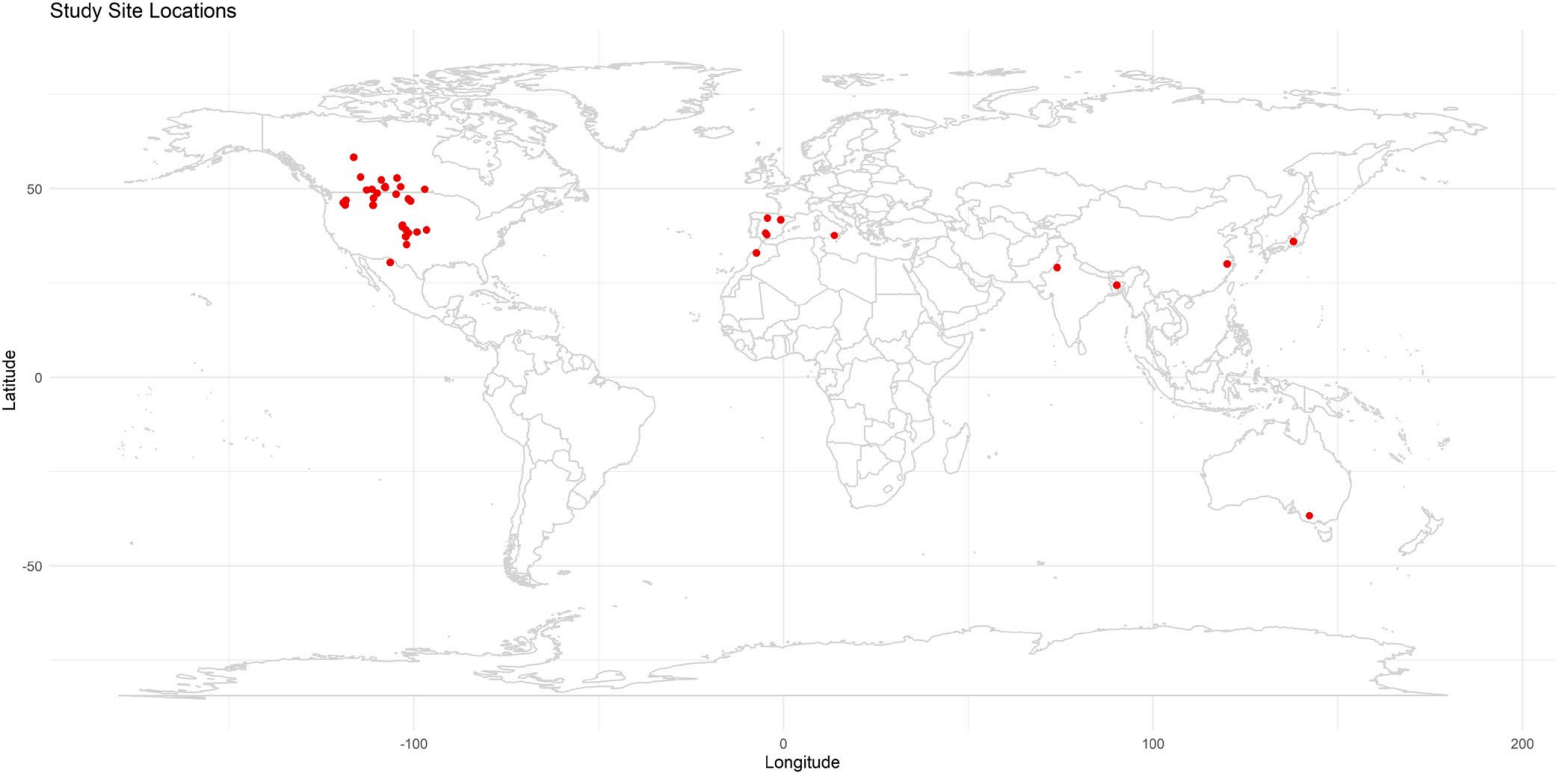
A map of study sites/locations globally. Map lines delineate study areas and do not necessarily depict accepted national boundaries.

A literature search was carried out using the Web of Science and Scopus databases. A broader search string was used as “(fallow) AND (soil organic carbon OR soil organic C OR SOC OR soil carbon OR soil C)”. With this search string, a total of 5834 entries were identified: 4486 from the Web of Science and 1348 from Scopus. There are many terms used for fallow in the literature. Only “rotational fallow” as part of regular crop rotation tailors the requisite. Thus, replacement of fallow with growing a crop shorter than maturity such as “cover crop” or “green manure” is excluded. We had a few additional criteria, including duration of field studies for longer than 4 years; soil depth of 15 cm or deeper; experimental design in which the difference in SOC stocks can be estimated between continuous annual crop rotations and crop rotations with fallow; and studies estimating SOC stocks or reporting SOC concentration and soil bulk density so that the stocks could be estimated. Out of the 5384 articles identified in the search, there were 27 papers with 97 paired comparisons from Canada, 26 articles with 73 paired comparisons from U.S.A. and 14 articles with 18 paired comparisons from other countries that met these criteria. The sites/locations from each individual publication are provided in Figure 1. A complete list of all studies with background information on citation, township, province/state, country, geo‐coordinates, climatic properties (mean annual temperature, precipitation, and potential evapotranspiration), soil taxonomy, soil texture, crop rotations, replicate, tillage, fallow frequency, soil sampling depth, SOC stocks with fallow‐based rotation and continuous cropping, net SOC stock change, and rate of SOC changes is provided in Table S1.

Soil textures for the study sites were either explicitly stated (e.g., fine texture) or presented as the proportion of sand, silt, and clay within the fine earth fraction (≤ 2 mm) of the soil. In the latter case, the particle size distribution was used to generate the soil texture that consists of three soil textural categories: coarse, medium, and fine.

The impact of eliminating bare summer fallow on SOC storage was quantified as:

(1) $$∆\mathrm{SOC}={\mathrm{SOC}}_{\mathrm{CC}}-{\mathrm{SOC}}_{\text{FALLOW}}$$

where ∆SOC is the net change in SOC storage between a continuous cropping system and a fallow‐containing cropping system (Mg C ha^−1^), SOC_CC_ is the amount of SOC in either a continuous cropping system or a crop system less frequency of bare fallow (Mg C ha^−1^), and SOC_FALLOW_ is the amount of SOC in the fallow‐containing cropping system (Mg C ha^−1^).

The relative difference in the SOC storage (RD_SOC_, % yr^−1^) between continuous cropping and fallow‐containing systems was calculated as:

(2) $${\mathrm{RD}}_{\mathrm{SOC}}=\frac{∆\mathrm{SOC}}{{\mathrm{SOC}}_{\text{BASE}}\times T}\times 100$$

where *T* is the duration of field experimental period (year).

The mean rate of SOC gain (SOCRATE, kg C ha^−1^ yr^−1^) between continuous and fallow‐containing cropping systems during the field experimental period was calculated:

(3) $$\text{SOCRATE}=\frac{∆\mathrm{SOC}}{T}$$

The net change in SOC storage between continuous cropping and fallow‐containing cropping systems can be assessed on either a per ha per year (kg C ha^−1^ yr^−1^) basis or a per ha per year/season of fallow reduction (kg C ha^−1^ yr^−1^ of fallow reduction). To account for variation in fallow frequency within the same site or among study sites, we calculated the rate of SOC gain/loss on a per ha and per year of fallow reduction (SOCRATE_EYFR_, kg C ha^−1^ yr^−1^):

(4) $${\text{SOCRATE}}_{\text{EYFR}}=\text{SOCRATE}\times \frac{1}{\mathrm{FF}}$$

FF (fraction) is the fallow frequency in a fallow‐containing cropping system. For instance, comparing ContW with FW, and ContW with FWWW, FF is 0.5 and 0.25, respectively.

The starting point of SOC stock should be taken into consideration if the SOC gain at the end of the study between crop systems with and without fallow is truly soil carbon gain or simply a reduction of SOC loss. However, most studies in this dataset did not provide the initial SOC stock with only a few exceptions (i.e., Campbell et al. 2005; Lin et al. 2023; López‐Bellido et al. 2020; Maillard et al. 2018; Sainju et al. 2015; Sombrero and de Benito 2010; Liebig et al. 2019). The absolute change in SOC stock between crop systems with and without fallow over time cannot be determined due to limited data from the original articles. Therefore, soil C change in this article can be referred to either absolute or relative gain in SOC and reflects some combination of less loss of SOC in a system that has been losing C over time, and/or a net removal of CO_2_ from the atmosphere with increasing storage in SOC.

Soil organic C storage under continuous and fallow‐containing systems for each site was statistically compared using paired T‐test through the SAS PROC TTEST procedure (SAS Institute 2001). For the net change in SOC on a per area or on a per area and year basis, statistical differences among soil types, texture classes, climatic factors [mean annual precipitation (P), potential evapotranspiration (PET), and ratio of P/PET], and duration of the management change were determined using the SAS PROC MIXED procedure (SAS Institute 2001). Differences in SOC were deemed significant between continuous and fallow‐containing systems, ∆SOC and the net change in SOC on per ha and year (kg C ha^−1^ yr^−1^ and kg C ha^−1^ yr^−1^ of fallow reduction) were considered significant when 95% confidence intervals did not overlap.

## 3 Results

Intensification of cropping systems with elimination of fallow resulted in a net change of SOC from −6.9 to 34.8 Mg C ha^−1^ with a mean of 3.2 ± 0.3 Mg C ha^−1^ that ranged from −290 to 1060 kg C ha^−1^ yr^−1^ with a mean of 180 ± 10 kg C ha^−1^ yr^−1^ or from −860 to 2490 kg C ha^−1^ yr^−1^ of fallow reduction with a mean of 440 ± 30 kg C ha^−1^ yr^−1^ of fallow reduction (Table 1). This wide range of differences in SOC stock changes might have resulted from variation in the duration of field experiments from 4 to 73 years, fallow frequency from 0% to 50% as well as variation associated with soil types and soil texture and different climates (Table 1). Other factors may also contribute to ∆SOC variation, including differences in methods of soil sampling, processing and analysis, spatial variability, corrections for equivalent mass, estimation of bulk density, and intrinsic SOC measurement variability.

**TABLE 1:** Soil organic carbon stock in continuous and fallow‐based cropping system (SOC_BASE_ and SOC_CC_), difference in SOC stock (∆SOC), relative SOC change and SOC gain annually based on either entire duration of cropping or per year of fallow reduction among soil texture classes.

Soil texture	Number of observation	Average duration of field studies (yr)	SOC_BASE_	SOC_CC_	ΔSOC	Relative SOC change	Rate of C change	
			Mg C ha^−1^	Mg C ha^−1^	Mg C ha^−1^	% yr^−1^	kg C ha^−1^ yr^−1^	kg C ha^−1^ yr^−1^ of fallow reduction
Coarse	15	14.5 (±1.4)	22.6** (±7.0)^b^	25.4 (±7.3)^b^	2.8 (±0.7)^ab^	1.3 (±0.3)^a^	180 (±40)^a^	430 (±90)^ab^
Medium	127	18.8 (±1.2)	34.7** (±1.9)^a^	37.5 (±2.0)^a^	2.8 (±0.3)^b^	0.6 (±0.1)^b^	170 (±20)^a^	410 (±40)^b^
Fine	45	24.7 (±3.0)	36.7** (±2.7)^a^	41.3 (±3.2)^a^	4.5 (±1.0)^a^	0.7 (±0.1)^b^	220 (±30)^a^	550 (±85)^a^

*Note:* Different letters between coarse, medium or fine are statistically significant at *p* < 0.05. ** indicates statistical significance through paired comparison between soil organic carbon stock in continuous (SOC_CC_) and fallow‐based cropping system (SOC_BASE_) at *p* < 0.05.

With continuous cropping, there was more carbon storage, averaging 4.5 Mg C ha^−1^ for the fine‐textured soil and 2.8 Mg C ha^−1^ for the medium and coarse‐textured soils (Table 1). On a relative basis, the intensification of cropping systems resulted in more SOC storage at a greater rate of 1.3% yr^−1^ for the coarse‐textured soil, compared with the medium‐ (0.6% yr^−1^) and fine‐textured (0.7% yr^−1^) soils (Table 1). On a per year basis, this resulted in increases in SOC storage of 220 (±30), 170 (±20), and 180 (±40) kg C ha^−1^ yr^−1^ for the fine‐, medium‐ and coarse‐textured soils, respectively (Table 1). On a per area and year of fallow or season reduction basis, there were gains in SOC stocks estimated at 550 (±85), 410 (±40), and 430 (±90) kg C ha^−1^ for the fine‐, medium‐ and coarse‐textured soils, respectively (Table 1).

The net SOC stock gain between crop systems with and without fallow was 2.4 (±0.5) Mg C ha^−1^ in arid regions (P/PET < 0.4), 3.7 (±0.4) Mg C ha^−1^ for the semiarid and subhumid region (P/PET: 0.4–0.8), and 3.0 (±1.4) Mg C ha^−1^ for the humid region (P/PET > 0.8), respectively (Table 2). These changes in SOC corresponded to a rate of 404 (±50), 472 (±46), and 347 (±165) kg C ha^−1^ yr^−1^ or season^−1^ of fallow reduction for the arid, semiarid and subhumid, and humid region, respectively (Table 2). More intensive cropping systems resulted in the highest net average gains of 6.7 (±1.9) Mg C ha^−1^ for Black Chernozem to 2.2 (±0.2) Mg C ha^−1^ for Brown Chernozem soils, equivalent to increases of 230 (±50) and 160 (±10) kg C ha^−1^ yr^−1^ on a per area and year basis (Table 2). Expressed in a per year of fallow reduction differences represented gains of 670 (±160), 445 (±100), and 410 (±40) kg C ha^−1^ for Black, Dark Brown and Brown Chernozem, respectively (Table 2).

**TABLE 2:** Soil organic carbon stock in continuous and fallow‐based cropping system (SOC_BASE_ and SOC_CC_), difference in soil organic carbon stock (∆SOC), and SOC gain annually based on either entire duration of cropping or per year of fallow reduction by soil type and ratio of mean annual precipitation (P) and potential evapotranspiration (PET).

Ratio of P/PET or soil zone	Number of observation	Average duration of field studies	SOC_BASE_	SOC_CC_	ΔSOC	Rate of C change	
			Mg C ha^−1^			kg C ha^−1^ yr^−1^	kg C ha^−1^ yr^−1^ of fallow reduction
*Range of P/PET ratio*							
< 0.4	69	17.2 (±2.0)	23.5** (±2.0)^c^	25.9 (±2.2)^c^	2.4 (±0.5)^b^	162 (±21)^bc^	404 (±50)^a^
0.4–0.8	113	22.1 (±1.4)	39.4** (±2.0)^b^	43.2 (±2.1)^b^	3.7 (±0.4)^a^	185 (±17)^b^	472 (±46)^a^
> 0.8	6	10.5 (±1.7)	61.8 (±9.5)^a^	64.8 (±9.9)^a^	3.0 (±1.4)^ab^	347 (±165)^a^	347 (±165)^a^
*Soil type* ^1^							
Brown Chernozem (Ratio of P/PET: 0.41 ± 0.10)	117	15.4 (±0.7)	27.3** (±1.2)^b^	29.5 (±1.2)^b^	2.2 (±0.2)^c^	160 (±10)^b^	410 (±40)^b^
Dark Brown Chernozem (Ratio of P/PET: 0.43 ± 0.12)	29	26.8 (±3.9)	41.2** (±4.2)^a^	45.4 (±4.7)^a^	4.2 (±1.0)^b^	200 (±40)^ab^	445 (±100)^ab^
Black Chernozem (Ratio of P/PET: 0.66 ± 0.07)	21	30.1 (±3.1)	43.7** (±3.8)^a^	50.4 (±4.6)^a^	6.7 (±1.9)^a^	230 (±50)^a^	670 (±160)^a^

*Note:* Different letters between soil zones or P/PET ratios are statistically significant at *p* < 0.05. **indicatestatistical significance through paired comparison between soil organic carbon stock in continuous (SOC_CC_) and fallow‐based cropping system (SOC_BASE_) within each soil type or P/PET ratio at < 0.01 and *p* < 0.05, respectively.

^1^ Soils from Canada and USA based on the Canadian Soil Taxonomy.

The duration of field studies also had an impact on SOC storage. With continuous cropping, there were gains of 2.2 (±0.3), 2.3 (±0.3), and 4.9 (±0.8) Mg C ha^−1^ for durations of less than 10 years, 11 to 20 years, and greater than 20 years, respectively. However, on a per area and year basis, the increase in storage rate declined on a per year of fallow reduction, from 615 (±70), 430 (±45), and 340 (±60) kg C ha^−1^ for durations of less than 10 years, 11 to 20 years, and greater than 20 years, respectively (Table 3).

**TABLE 3:** Soil organic carbon stock in continuous and fallow‐based cropping system (SOC_CC_ and SOC_BASE_), difference in SOC stock (∆SOC), and SOC gain annually based on either entire duration of cropping or per year of fallow reduction with various duration of field experimental period.

Duration	Number of observation	Average duration of field studies (yr)	SOC_BASE_	SOC_CC_	ΔSOC	Rate of C change	
			Mg C ha^−1^			kg C ha^−1^ yr^−1^	kg C ha^−1^ yr^−1^ of fallow reduction
Less than 10	44	8.0 (±0.3)	31.3** (±2.6)^b^	33.5 (±2.7)^b^	2.2 (±0.3)^b^	260 (±30)^a^	615 (±70)^a^
11–20	77	13.2 (±0.3)	32.1** (±2.9)^b^	34.4 (±3.0)^b^	2.3 (±0.3)^b^	170 (±20)^b^	430 (±45)^b^
Great than 21	67	35.5 (±2.0)	38.5** (±2.3)^a^	43.3 (±2.6)^a^	4.9 (±0.8)^a^	140 (±20)^b^	340 (±60)^b^

*Note:* Different letters among various duration of field studies are statistically significant at *p* < 0.05. ** indicatestatistical significance through paired comparison between soil organic carbon stock in continuous and fallow‐based cropping system (SOC_CC_ and SOC_BASE_) within each duration of field studies at < 0.01 and *p* < 0.05, respectively.

## 4 Discussion

The rate of change in SOC storage on a per area and year basis associated with the intensification of cropping systems from this study was comparable with the previous findings of 160 kg C ha^−1^ yr^−1^ derived from 10 studies with 19 comparisons without considering the exact change in frequency of fallow (VandenBygaart et al. 2003). Unlike cover crops, a simple reduction of summer fallow has not received wide attention as a mitigation option globally (Griscom et al. 2017; Fargione et al. 2018; Drever et al. 2021; Minasny et al. 2017), likely due to the limited areas of opportunity, such as in regions with practices related to dry weather agriculture like the Great Plains of North America. Poeplau and Don (2015) conducted a meta‐analysis to derive a carbon response function and reported that cover crops in crop systems increased SOC with an annual change rate of 320 ± 80 kg C ha^−1^ yr^−1^ in a mean soil depth of 22 cm. The rate of SOC gain on a per ha and year or season of fallow reduction observed in our study is greater than with cover cropping. Models encapsulate current knowledge of SOC dynamics and are frequently used to estimate and extrapolate changes in SOC. Several empirical models have been developed with a varying degree of success for simulating SOC stock changes by comparing crop rotations with or without summerfallow (Campbell et al. 2001; Liang et al. 2005; Shrestha et al. 2013). Smith et al. (2012) employed three process‐based models (CENTURY, DAYCENT, and DNDC) and the CAMPBELL empirical model to simulate SOC change with observations from 14 residue removal experiments within the temperate climate areas of Canada and the Midwestern USA. They reported that residue removal effects on SOC were more likely to be observed (i) with greater rates of residue removal, (ii) after longer periods, and (iii) with greater rates of fertilization.

The results of this analysis reveal that soil texture, soil type, climate, and duration of the management practice play a major role in influencing the SOC storage from cropping system intensification associated with reducing bare‐fallow. Fine‐textured soils had the greatest amount of SOC accrual (4.5 Mg C ha^−1^) associated with the reducing bare‐fallow, compared with the coarse‐ and medium‐textured (2.8 Mg C ha^−1^) soils (Table 1). Coarse‐textured soils had a greater rate of SOC gain on a relative basis (1.3% yr^−1^) than the medium‐ (0.6% yr^−1^) and fine‐textured (0.7% yr^−1^) soils (Table 1). Liang et al. (2020) reported an increase of 0.23 to 1.30% yr^−1^, depending on length of management change on the Canadian prairies with a change from conventional tillage to no‐till that appears to be proportional with the observations of cropping system intensification. The mean rate of SOC gain for the fine‐textured soils associated with the intensification of cropping system was significantly higher than the medium‐textured and the coarse‐textured soils (Table 1), implying that the coarse‐ and fine‐textured soils are more responsive to reductions in bare‐fallow. Liang et al. (2020) reported that no‐till increased SOC by 580 kg C ha^−1^ yr^−1^ in the coarse‐textured soils, 200 kg C ha^−1^ yr^−1^ in the medium‐textured soils, and 620 kg C ha^−1^ yr^−1^ in the fine‐textured soils on the Canadian prairies. Other studies also suggest that the fine‐textured soils on the Canadian prairies are more responsive to practices that enhance crop yields such as no‐till and cropping system intensification (McConkey et al. 1996; VandenBygaart and Liang 2024). No‐till and intensification of cropping system also reduce wind erosion especially for coarse‐textured soils (McConkey et al. 2003). Bare summer fallow may also accelerate soil C losses by erosion, but these losses are localized, often resulting in redistribution of C across the landscape rather than release to the atmosphere (Gregorich et al. 1998).

The ratio of P to PET is a key indicator of aridity in a region. The increased levels of SOC storage associated with the intensification of cropping systems in semiarid, subhumid, and humid regions, compared with the arid region, might have resulted from a reduced moisture deficit and greater crop productivity (Liang et al. 2005). The intensification of cropping systems resulted in a greater net gain of SOC storage for the more productive Black Chernozem than in the Dark Brown and Brown Chernozem (Table 2). There is a clear linkage between the ratio of P/PET and soil type (i.e., Brown, Dark Brown, and Black Chernozem in Canada and the USA) (Table 2).

The time of adoption is a key determinant of SOC accrual with cropping system intensification. The rate of SOC stock change, expressed in either kg C ha^−1^ yr^−1^ or kg C ha^−1^ yr^−1^ of fallow reduction, was significantly higher within a period of less than 10 years whereas there was no difference in the rate of SOC stock change between 11 and 20 and greater than 20 years (Table 3).

Liang et al. (2020) reported a similar result for no‐till adoption on the Canadian prairies with rates of 740 kg C ha^−1^ yr^−1^ between 3 and 10 years that declined to 260 kg C ha^−1^ yr^−1^ between 11 and 20 years, and 95 kg C ha^−1^ yr^−1^ for a period of greater than 21 years. This basic comparison shows that the rate of SOC gain corresponding to a more intensive cropping system is similar to that of switching from conventional tillage to no‐till, particularly within 10 years of management change. However, our results differ with the further reduction of the rate of SOC gain occurring between 11 and 20 years and greater than 20 years from the no‐till finding with little change occurring in the rate of SOC gain with more intensive cropping systems after 11 years. It should be recognized that the rate of SOC gain with the reduction of bare summer fallow is mainly a direct result of crop residual C input while the rate of SOC gain under no‐till responded to climate and soil texture, but to a lesser extent to crop residual C input (Liang et al. 2020; VandenBygaart and Liang 2024). Based on current understanding of SOC encapsulated in many different models, change in SOC after a change in management is rapid initially and converges to a steady state within one to several decades, though with possible slow gradual change of recalcitrant fractions over centuries. One of the objectives from summerfallow is to conservate soil moisture that might be favored for soil C decomposition by microbes (Campbell, Zentner, Liang, et al. 2000), but the net impact on soil C loss through increased soil moisture under summerfallow, compared with continuous cropping in this dataset cannot be determined. Declining rates with time are expected based on models and evident in most studies where a change in cropping practice is measured periodically over a long period (Liang et al. 2020). To our knowledge, our study is the first report of declining rates of SOC gain over time in response to the change in fallow reduction through a meta‐analysis.

Soil C changes can occur in both the top and subsurface soil horizons (e.g., Poeplau and Don 2015) and consequently soil sampling depth is also a complicating factor. However, in the semiarid climate where fallow is more often practiced, tillage is usually shallow at no more than 15 cm and most of SOC changes are expected to occur near the soil surface given the moisture limitations on microbial activity. Typically, absolute differences in SOC among treatments increase with the depth, but significant differences decline because total mass of C measured and its variability increase much more than the difference among treatments (Ellert et al. 2001; Kravchenko and Robertson 2010). Significant differences are often observed only in the surface 7.5 or 10 cm, if at all (Campbell, Zentner, Selles, et al. 2000; Sherrod et al. 2003). Therefore, most of the literature dealing with the intensification of cropping systems have focused on the 0‐ to 15‐cm depth, and so we restricted our analysis to the topsoil layer (Table S1). Future studies should further investigate effects deeper in the soil with sampling to evaluate the significance of management impacts in subsurface horizons compared to the topsoil.

There were 31 articles that include tillage as a treatment factor in the dataset in addition to cropping systems with and without fallow (Table S1). There are two problems with the data: (1) unbalanced experimental designs (i.e., for fallow‐based cropping system with no‐till and reduced tillage whereas for continuous cropping system with no‐till, reduced, and conventional tillage, 10 articles), and (2) a lack of SOC data for a combination of cropping systems and tillage practices, only showing means of SOC data for cropping systems or tillage practices and an indication of absence in interaction (5 articles). Of all these studies, there are only 18 studies that presented SOC stocks for a combination of tillage and crop rotations. There are other issues including non‐replicated results and SOC stocks measured with varying soil depths across sites and studies. We analyzed the data statistically but failed to determine any significant interaction between cropping system and tillage on soil C stock change. The relationship between tillage and fallow frequency deserves more attention in the future as more studies are completed providing insight into this topic.

There are only 14 articles with 18 observations outside of U.S.A. and Canada (Table S1). The observations outside of North America are sparse and six observations have a ratio of precipitation to potential ET > 1 with a distinctive wetter and dryer season (Alam et al. 2017; Higashi et al. 2014; Wang et al. 2023; Chen et al. 2016). The intensification of cropping systems, outside of U.S.A. and Canada, resulted in an overall mean net change of SOC by 3.2 (±0.6) Mg C ha^−1^, corresponding to 240 (±65) kg C ha^−1^ yr^−1^ or 380 (±90) kg C ha^−1^ season^−1^ of fallow reduction. These rates of SOC gains were similar to the results obtained from USA and Canada. It should be noted that the results observed from India, China and Japan involved two or three crops annually (Table S1). Globally, the intensification of cropping system gained, on average, 3.1 (±0.4) Mg C ha^−1^, corresponding to 185 (±18) kg C ha^−1^ yr^−1^ or 462 (±42) kg C ha^−1^ yr^−1^ or season^−1^ of fallow reduction. It is important to recognize that the mechanism of increased soil C storage with the reduction of fallow is an increased crop residue returned to the soil through increased crop productivity, that is independent of regions. This suggests that crop residue input is the main driver dictating soil C stock changes. Our results are consistent with a recent concept of photosynthetic limits on carbon sequestration in croplands, stressing the linkage between crop residue input and soil C storage (Janzen et al. 2022).

**FIGURE 2:**
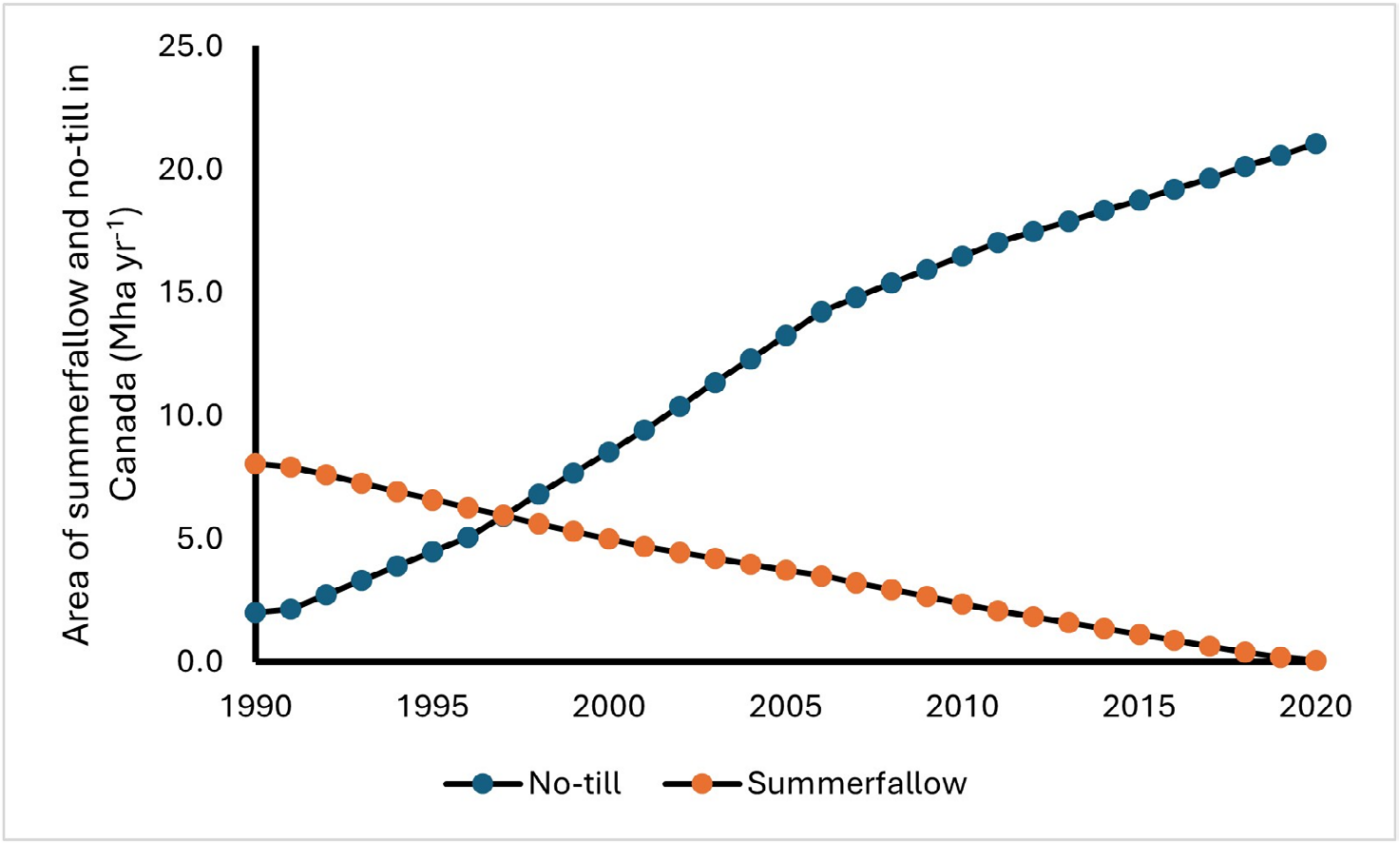
Summer fallow and no‐till area from 1990 to 2020 in Canada (Data source: Environment and Climate Change Canada, 2024).

There is a strong link between adoption of no‐till and reduction of summerfallow on the Canadian prairies. The rapid adoption of no‐till from 1990 (1.9 Mha or 6%) to 2016 (18.2 Mha or 64%) was also accompanied by a steep decline in summerfallow (8 Mha) during the same period on the Canadian prairies (ECCC 2024). In contrast, the adoption of no‐till on the Central and Northern Great Plains of USA is slower. Based on the 2017 Agricultural Census data, 37% of reported acres in the United States were under both continuous no‐till and rotational no‐till (Chen et al. 2022), and 28% under continuous no‐till (U.S. Department of Agriculture 2024). It is not clear why the adoption rates vary between the two countries. The Canadian prairies provide an example of the implementation of a significant management change in which the area of bare summer fallow decreased linearly from 8 Mha in 1990 to 0.1 Mha in 2020 (Figure 2). Bare summer fallow was widely practiced, but with the adoption of no‐till in the prairie provinces of Alberta, Saskatchewan, and Manitoba, the area of bare summer fallow has declined. During the same period, the area of no‐till increased from 2 Mha in 1990 to 14 Mha in 2006 and approached 21 Mha in 2020 (Figure 2). Several management practices have contributed to the reduction in bare summerfallow on the Canadian prairies including: (1) replacing fallow with drought‐tolerant crops such as millet, chickpeas, flax or mustard (Zentner et al. 2002; Gan et al. 2011), (2) using conservation tillage or no‐till systems (Zentner et al. 2002; Smith et al. 2016), (3) maximizing soil moisture capture and retention (Cutforth et al. 2011; Cutforth 2013), (4) inclusion of perennials or forage crops in the cropping system (Bruce et al. 1999; Bremer et al. 2002), (5) integrating livestock where possible (Martens and Entz 2011), and (6) adapting precision agriculture and soil testing (Pennock 2005). All these practices serve multiple purposes to preserve soil moisture, improve soil structure, promote nutrient cycling, and maintain the economic viability of the farming operation over time.

Based on the annual area of fallow reduction in Canada from 1990 to 2022 and a mean rate of soil C stock change of 470 kg C ha^−1^ yr^−1^of fallow reduction, a cumulative gain of SOC, with either removals of CO_2_ from the atmosphere and/or reduced SOC losses of 66.3 Mt C or 243 Mt CO_2_eq has been observed in agricultural soils since 1990 (Table S2).

Globally, the area of cropland in bare‐fallow, according to FAO statistics, amounted to 203 Mha in 2001, 189 Mha in 2010, and 176 Mha in 2020, respectively (Table 4). The area of fallow has decreased by 7% between 2001 and 2010 and another 6% between 2011 and 2020. Vittis et al. (2021) estimated global cropland area of 1500 Mha, and thus the proportion of fallow area was still approximately 11.7% in 2020. The countries with most areas of bare‐fallow in 2020 were India (26.2 Mha), Pakistan (15.2 Mha), China (14.8 Mha), Indonesia (13 Mha), Brasil (9.3 Mha), Russian Federation (7.2 Mha), United States of America (6.9 Mha) and Australia (4.9 Mha) (Table 4). As an exercise, let's assume total elimination of fallow within 30 years (a mean of 3.3% yr^−1^, the same rate of bare‐fallow reduction occurred in Canada) with promotion of no‐till to address moisture limitations in these semiarid climates and a mean rate of soil C increase of 440 kg C ha^−1^ yr^−1^ of fallow reduction, there would be a cumulative gain of SOC storage for a period of 20 years amounting to 0.54 Gt C or 2.0 Gt CO_2_eq globally, an average of roughly 100 Mt CO_2_eq annually (Table S3).

**TABLE 4:** Global area of cropland under fallow and relative change for 2001, 2010 and 2020.

Country	2001	2010	2020	2010–2001	2020–2010
	Mha			%	
Afghanistan	5.2	4.3	4.0	−16.8	−7.4
Algeria	3.7	3.3	3.0	−12.5	−7.5
Argentina	4.3	3.3	2.3	−23.2	−30.7
Australia	4.6	4.4	4.9	−2.4	10.1
Brazil	9.6	9.3	9.3	−2.5	−0.3
Canada	4.7	2.4	0.7	−49.4	−70.5
China	17.6	16.4	14.8	−6.8	−9.5
India	25.0	26.8	26.2	7.2	−2.5
Indonesia	9.3	11.7	13.0	24.9	11.4
Iran (Islamic Republic of)	4.6	4.1	4.5	−11.2	10.6
Kazakhstan	7.1	4.5	3.9	−36.3	−14.1
Mexico	8.4	6.7	4.0	−19.7	−40.9
Morocco	2.3	2.0	1.5	−12.4	−25.5
Niger	2.0	2.0	2.2	−0.2	7.8
Nigeria	4.8	4.5	4.3	−7.7	−2.4
Pakistan	15.9	13.5	15.2	−15.2	12.4
Peru	2.5	2.9	2.7	15.4	−5.1
Russian Federation	10.6	10.6	7.2	−0.9	−32.0
Saudi Arabia	2.4	2.2	2.5	−6.8	11.8
Spain	3.5	3.8	2.9	8.5	−24.7
Türkiye	4.9	4.2	3.2	−13.5	−25.3
United States of America	6.8	6.0	6.9	−12.1	15.3
All other countries^a^	43.7	40.1	37.0	−8.1	−7.9
Total	203.4	189.0	176.0	−7.1	−6.9

*Note:* Data source: Food and Agriculture Organization of the United Nations (https://www.fao.org/faostat/en/#data/RL). Access data: December 12, 2025.

^a^ Sum of all countries with less than 1% of contribution each.

## 5 Conclusion

Most of the studies eliminating or reducing bare‐fallow frequency cited in this study have been conducted in the Central and Northern Great Plains of North America, and further research is needed in other regions. Regardless, this is the first study that provides the rate of SOC gain with cropping system intensification based on the number of years of bare‐fallow reduction, which is a key consideration for determining the level of SOC storage with adoption of this climate smart practice. The results of our study show that soil types, soil texture, climate, and the time since adoption of this climate smart soil practice are the main drivers of SOC change with the intensification of cropping systems. These factors must be considered in the development of either national or regional SOC models associated with estimating the impact of reducing bare summer fallow. The results of this study further suggest that detailed stratification of soil types, soil texture, aridity index, and duration of crop intensification is required for more accurate estimates of SOC storage.

The intensification of cropping systems by eliminating or reducing the frequency of bare‐fallow has effectively created an additional 8 Mha of fertile cropland, contributed to improved crop productivity, and enhanced SOC storage in Canada. It is worthy of noting that the elimination of bare‐fallow may accompany adoption of no‐till and further contribute to changes in SOC. Considering these findings, a significant reduction of fallow area is feasible on a global scale with an estimate of SOC change at 0.54 Gt C or 2.0 Gt CO_2_eq for a period of 20 years and could contribute to national efforts limiting warming to 2^o^C through the Paris Agreement. Contrary to some proposed nature‐based climate solutions, global reduction of bare‐fallow, stacked with other effective environmental stewardship practices, such as careful nutrient management, is a climate change mitigation opportunity that may increase profitability with improved soil water holding capacity and higher levels of SOC, as well as increase global food production for a growing population. We stress that, however, increased cropping intensity might have impacts on emissions associated with nutrient management (e.g., nitrogen) and thus it is important to pair increased cropping intensity with effective nitrogen management.

## Author Contributions

**Chang Liang:** conceptualization, data curation, formal analysis, investigation, methodology, writing – original draft, writing – review and editing. **Bert VandenBygaart:** conceptualization, formal analysis, investigation, methodology, writing – review and editing. **Stephen Ogle:** conceptualization, investigation, methodology, writing – review and editing. **Douglas MacDonald:** conceptualization, project administration, writing – review and editing. **Gabriel Dias Ferreira:** data curation, investigation, methodology, writing – review and editing.

## Conflicts of Interest

The authors declare no conflicts of interest.

## Supporting information


**Table S1:** Background information on field studies of crop rotation including frequency of fallow, geographic location.
**Table S2:** Soil carbon sequestration as estimated from annual reduction of fallow from 1990 to 2022 in Canada.
**Table S3:** Estimating the amount of annual soil organic (SOC) sequestration by assuming a fixed rate of fallow reduction (3.3% yr^−1^) and a mean rate of SOC gain (0.44 Mg C ha^−1^ yr^−1^ of fallow reduction) from 2021 to 2040 globally.

## Data Availability

The data that supports the findings of this study is available in the Supporting Information of this article.
