# Supplementary material for: Global Potential to Increase Soil Carbon Storage by Reducing Rotational Fallow in Semiarid Regions
Source: Glob Chang Biol. 2025 Dec 26;32(1):e70672. doi: 10.1111/gcb.70672 (PMC12741790; doi:10.1111/gcb.70672)
Supplement: Supplementary file 1 — Table S1: Background information on field studies of crop rotation including frequency of fallow, geographic location. Table S2: Soil carbon sequestration as estimated from annual reduction of fallow from 1990 to 2022 in Canada. Table S3: Estimating the amount of annual soil organic (SOC) sequestration by assuming a fixed rate of fallow reduction (3.3% yr−1) and a mean rate of SOC gain (0.44 Mg C ha−1 yr−1 of fallow reduction) from 2021 to 2040 globally. [file GCB-32-e70672-s001.docx]

Supporting Information

SI Table 1

Background information on field studies of crop rotation including frequency of fallow, geographic location, climatic factors, soil taxonomy, soil texture, study duration, soil sampling depth, soil organic carbon stock (SOC), net change in SOC, and rate of SOC change

SI Table 2.

Soil carbon storage as estimated from annual reduction of fallow from 1990 to 2022 in Canada.

SI Table 3.

Estimating the amount of annual soil organic (SOC) storage by assuming a fixed rate of fallow reduction (3.3% yr^-1^) and a mean rate of SOC gain (0.49 Mg C ha^-1^ yr^-1^ of fallow reduction^)^ from 2021 to 2040 globally.
